# Patient-reported joint count in juvenile idiopathic arthritis: the reliability of a mannequin format

**DOI:** 10.1186/1546-0096-12-S1-P175

**Published:** 2014-09-17

**Authors:** Janneke Anink, Maryanne Dijkstra, Philomine PA van Pelt, Johanna M W Hazes, Lisette W A van Suijlekom-Smit

**Affiliations:** 1 Paediatric Rheumatology, Erasmus MC Sophia Children's Hospital, Rotterdam, Netherlands; 2 Rheumatology, Erasmus MC, Rotterdam, Netherlands

## Introduction

Juvenile idiopathic arthritis (JIA) is a common chronic disease, requiring regular monitoring. Patient-reported outcomes can assist monitoring, may promote patient self-management and can be useful in epidemiological surveys.

## Objectives

To evaluate reliability of a mannequin-format patient-reported joint-count in JIA, and to detect changes in agreement at a follow-up visit.

## Methods

JIA patients aged 12-21 marked joints with active arthritis on a mannequin before their regular clinic visit. The physician performed a joint-count without having seen the patient’s assessment. For two subsequent clinic visits, agreement between the physician and patient-reported joint-counts was assessed using Intraclass Correlation Coefficient (ICC) and kappa statistics. The ability of the patient-reported joint-count to discriminate between active and inactive disease was evaluated using positive and negative predictive values. Sensitivity to change was estimated using Pearsons’s rho and standardized response mean (SRM).

## Results

75 JIA patients were included. In general, patients had a low number of active joints (median 1 joint, indicated by the physician). ICC was moderate (0.61) and kappas ranged from 0.3-0.7. At the follow-up (n= 53), kappas were similar; the ICC was 0.19. When a patient scored 0 joints, the physician confirmed this in 93-100%. When the patient marked ≥ 1 joints, the physician confirmed arthritis in 59-76%. Sensitivity to change was moderate (Pearson’s rho: 0.44, p=0.001, SRM in worsening patients: 0.67).

## Conclusion

Agreement between physician and patient on joint-counts was reasonable. Untrained patients tended to overestimate presence of arthritis when they marked active joints on a mannequin-format joint-count. When the patient indicated absence of arthritis, the physician usually confirmed this. The sensitivity to change was moderate for patients who worsened over time. The agreement did not improve at follow-up; future research should focus on the possibility of achieving this through training. For now, the patient-reported joint-count cannot fully replace the physicians’ joint-count in clinical practice; it may be used in epidemiological studies with caution.

## Disclosure of interest

None declared.

